# Experimental Study on Longitudinal Acceleration of Urban Buses and Coaches in Different Road Maneuvers

**DOI:** 10.3390/s23063125

**Published:** 2023-03-15

**Authors:** Damian Frej, Paweł Grabski, Rafał S. Jurecki, Emilia M. Szumska

**Affiliations:** Faculty of Mechatronics and Mechanical Engineering, Kielce University of Technology, al. Tysiąclecia Państwa Polskiego 7, 25-314 Kielce, Poland

**Keywords:** vehicle dynamics and stability, longitudinal acceleration, vehicle testing

## Abstract

A vehicle’s longitudinal acceleration is a parameter often used for determining vehicle motion dynamics. This parameter can also be used to evaluate driver behavior and passenger comfort analysis. The paper presents the results of longitudinal acceleration tests of city buses and coaches recorded during rapid acceleration and braking maneuvers. The presented test results demonstrate that longitudinal acceleration is significantly affected by road conditions and surface type. In addition, the paper presents the values of longitudinal accelerations of city buses and coaches during their regular operation. These results were obtained on the basis of registration of vehicle traffic parameters in a continuous and long-term manner. The test results showed that the maximum deceleration values recorded during the tests of city buses and coaches in real traffic conditions were much lower than the maximum deceleration values found during sudden braking maneuvers. This proves that the tested drivers in real conditions did not have to use sudden braking. The maximum positive acceleration values recorded in acceleration maneuvers were slightly higher than the acceleration values logged during the rapid acceleration tests on the track.

## 1. Introduction

Urban buses and coaches are some of the most important collective transport means. An urban bus is a vehicle planned to transport numerous people over small distances. In addition to seating, city buses have room for standing passengers, spacious and comfortable aisles among seats, and spacious entrance doors. An urban bus is adapted to move in urban conditions characterized by frequent accelerations and braking, and thereby its maximum speed usually is not very high. Passengers in these vehicles do not have seat belts at their disposal and may therefore be at risk of injury in driving situations that require dynamic driver responses.

Coaches are vehicles that usually carry out passenger transport at long distances, including interurban and international routes. These vehicles usually do not use traditional stops and are most often not restricted by rigid time schedules. Coaches are only equipped with seats and have dedicated spaces for luggage. Coaches are also characterized by higher passenger comfort than urban buses. Passengers have at their disposal comfortable, adjustable seats with armrests and tables, air-conditioning, and monitors for watching movies, and even fridges and a bar with hot drinks. Coach passengers should use the seat belts installed at each seat while driving.

Urban buses and coaches differ in terms of dynamic driving properties. Acceleration is one of the parameters that enable the testing of differences between a vehicle’s dynamic parameters. Longitudinal acceleration is directly related to acceleration and braking maneuvers. Acceleration and deceleration values depend on the maneuver’s intensity applied by the driver and vehicle type. The recording of longitudinal acceleration during experimental testing can be done with the use of various devices, including a 3-axis accelerometer, acceleration sensors with a GPS module, and recorders that read data from the CAN bus using OBD connections. Lately, it is becoming increasingly popular to use smartphones with dedicated applications. However, they are characterized by various measurement accuracies.

Accelerometers are widely used in vehicle dynamics studies. In vehicle monitoring, it is necessary to differentiate between acceleration, deceleration, and lateral acceleration, because these data can be used to effectively identify and classify vehicle maneuvers. Three-axis accelerometers are used to measure vehicle acceleration relative to three perpendicular coordinate axles. Examples of using three-axis accelerometers for vehicle dynamics testing can be found in [1,2,3]. Currently, there are increasing numbers of studies in which accelerometers are mounted in wheel rims [4,5,6].

The method of collecting acceleration profiles using GPS sensors allows for automatic and continuous recording of acceleration values while driving without the need to modify the vehicle’s design or to design the experiment in a specific way. Sensors with a GPS module provide information about the vehicle’s instantaneous position, instantaneous speed, instantaneous acceleration values, distance traveled, and travel time. Examples of this method’s application in recording acceleration values and other dynamic parameters of a vehicle while driving are presented, among others, in [7,8,9,10].

In many papers, the dynamic properties of a vehicle in real conditions were evaluated using data derived from a CAN (controller area network) bus. A CAN bus is a two-wire network with real-time data transmission. Each electrical module of the vehicle is monitored and controlled using one or more sensors that provide notification to and interoperate with the main control unit (MCU). Microcontrollers (that operate the sensors) communicate with the MCU and between one another using typical communication standards based on a bus, such as the CAN bus. The data that can be obtained from the CAN bus include longitudinal and lateral acceleration, brake pedal use, accelerator pedal use, or engine speed. Examples of using data derived from a CAN bus to test the dynamic parameters of a vehicle can be found in [11,12,13,14].

Another popular method of collecting vehicle driving parameters is to use an OBD connection that plugs into a dedicated port on the vehicle. As mentioned earlier, the vehicle’s electronic units communicate with one another using a network (CAN bus or similar) to monitor and transmit data. Vehicle dynamic parameters are derived from a microcontroller that uses the OBD protocol (on-board diagnostics). OBD is the vehicle’s reporting and diagnostic function that allows the vehicle operator or technician to access the condition of engine subsystems. Vehicles usually have an external port that enables the downloading of values of selected parameters and reading errors, as well as identifying the error code source. Access to the monitored values is provided by using OBD scanners. OBD connections can be used to record lateral acceleration and longitudinal acceleration, engine speed, acceleration and braking pedal position, or fuel consumption. The method of collecting data using an OBD connection to obtain the vehicle’s dynamic parameters is presented in studies [15,16,17,18].

Using a smartphone as a device to record acceleration profiles is relatively inexpensive; however, the data captured are limited to features allowed by the smartphone. Common sensors provided by a smartphone include accelerometers, gyroscopes, and global positioning systems (GPS). Based on the possibility to record the vehicle’s acceleration and instantaneous position, dedicated applications were developed that constitute an element of the measurement system. For example, in papers [19,20,21,22], it is possible to find an application that determines the driver’s profile using a statistical model based on the recorded longitudinal and lateral acceleration values while driving.

Acceleration profiles are used to analyze and assess many aspects related to vehicle operation and safety. Many papers present results of experimental testing aimed at determining the relation between acceleration momentum and speed [23,24,25,26,27]. In the literature, it is possible to find a comparison of longitudinal acceleration values recorded by several types of vehicles. Analyses conducted in works [28,29,30] show that accelerating a vehicle to a higher desired speed requires a longer acceleration time and a longer acceleration distance. Vehicles braking from a higher initial speed show longer deceleration times, longer deceleration distances, and lower deceleration values. This is important in terms of the active safety of buses.

Due to the specificity of urban traffic conditions and high traffic intensity, public transport can be much more often involved in accidents or collisions. It is estimated that several hundred accidents involving buses take place each year. For example, in 2020, 189 accidents involving urban buses, 6 fatalities, and 232 wounded were recorded in Poland. In the case of other buses (coaches), 34 accidents involving 4 fatalities and 73 wounded were recorded. Traffic incidents that take place on different road types and in different atmospheric conditions are subject to evaluations of road traffic experts aimed at determining the causes of the traffic accident. These evaluations require obtaining the limit values of acceleration recorded during sudden braking and acceleration, which can be applied as a result of the tests described in this paper. The necessity of updating the related data must also be noted. This is related to the continuous improvement of vehicle braking systems, wheel slip control systems during acceleration and braking (e.g., BAS, ABS, ASR, TCS, ATC, ESP), and the use of, e.g., electric drive systems.

Longitudinal acceleration studies and analyses obtained during testing conducted in the normal operation of these form of transport enable the detection of dangerous driver behavior when driving a bus. When analyzing the data in a longer time interval, it becomes possible to determine the probability of accidents and collisions caused by a driver’s dangerous driving. The acceleration and deceleration characteristics can point to different driver danger behaviors, thereby making it possible to eliminate them. The results described in studies [31,32,33] established that each driver uniquely perceives the environment and reacts subjectively to changing road conditions, which is reflected in acceleration values. Several studies have been reported in the literature in which driving style and driver behavior are determined by longitudinal acceleration profiles. Better and more effective brakes in a vehicle enable faster deceleration to avoid an accident, on one hand, but on the other hand, higher deceleration can cause dangerous situations among the vehicle’s passengers [34,35]. Considering the specificity of bus designs, e.g., possible standing places, such tests can provide an opportunity to determine situations in which passengers can feel discomfort during travel. Many studies and scientific papers confirm that longitudinal acceleration is one of the factors that determine the comfort of passengers in urban buses and coaches. Papers [36,37,38,39] present the results of a study with the evaluation of acceleration values considered to be uncomfortable by seated standing and moving passengers on the bus, registered at different speeds and traffic conditions.

A literature review allowed for the observation that there are not very many studies that demonstrate the real longitudinal acceleration of urban buses and coaches.

The objective of this study is to present methodology to determine the extreme values of longitudinal accelerations recorded in test conditions and then compare them with the acceleration values obtained in driving conditions in real traffic conditions. Extreme longitudinal acceleration values are expressed as acceleration and deceleration ranges. These values were determined for city buses and coaches during sudden braking and acceleration maneuvers. The testing conducted as part of this research project featured two stages. In the first stage, special test sections were used to conduct sudden braking maneuvers and intense acceleration maneuvers from a standing start. This made it possible to determine the maximum acceleration and deceleration values under laboratory conditions. In the second stage, longitudinal acceleration was analyzed while driving the test vehicles in real road traffic conditions. The acceleration values collected under real conditions could be compared to the maximum values set in the track tests. The presented results are part of a project aimed at developing an application to determine and assess the driving style of drivers of several types of vehicles.

Longitudinal acceleration is one of the parameters used to examine a driver’s driving style. These parameters are important in the case of urban buses and coaches due to passenger comfort. Reliable deceleration and acceleration values of vehicles are important for car experts. Acceleration values differ depending on the vehicle type. Programs intended for complex evaluation of road incidents utilize sophisticated vehicle models aimed at conducting a space and time analysis that covers any type of moving object. Many of these programs require the specification of the vehicle’s longitudinal acceleration. A literature review allowed for the observation that not many publications demonstrate updated results of longitudinal acceleration tests conducted on urban buses and coaches. Obtaining true and reliable longitudinal acceleration values requires appropriate laboratory tests using specialized equipment.

This paper shows the results of part of the research carried out as part of a project to determine the driving style of drivers of different types of vehicles (including urban buses and long-distance coaches) under different road conditions. The maximum and minimum values of longitudinal acceleration determined in the test conditions were the basis for the initial evaluation of the drivers. High acceleration values recorded by the driver during regular driving were considered potentially dangerous and risky behavior. The ultimate goal of the project, of which the presented results are part, was to use longitudinal acceleration as one of the factors determining driver behavior. Thanks to this, it was possible to identify drivers with dangerous behavior that could lead to dangerous road incidents in the future.

In this paper, Section 2 shows the methodology of the conducted research. The tests of accelerations were carried out on the track and in a real-world driving condition. The parameter recorded during the tests was longitudinal acceleration. Section 3 presents the acceleration values recorded during rapid acceleration and braking maneuvers as well as the acceleration values recorded while driving in real traffic conditions. Section 4 discusses the differences between the acceleration values obtained by the city bus and coach in track tests and the acceleration values collected during the normal operation of the vehicles. The obtained results were compared with the results of similar tests presented in the literature. Finally, Section 5 presents the main conclusions from the analyzes carried out in the study.

## 2. Research Methodology

In this paper, an experimental study of vehicles was performed. The parameter investigated was longitudinal acceleration, recorded using specialized measuring equipment. The experimental research was carried out in two stages. The first stage was carried out under specific measurement conditions on specially prepared measuring sections on the track. The second was carried out in real conditions during regular vehicle operation. The data collected during the experimental tests were then statistically analyzed using the Statistica program (StatSoft, version 13).

As mentioned above, buses differ in terms of dynamic properties during driving based on their intended purpose and design. It was therefore decided to conduct testing of longitudinal acceleration values for two specific types of buses, i.e., an urban bus and a coach. These vehicles differ, due to their purpose, in their dynamic performance. The testing was conducted in two stages.

In the first stage, measurements were made during acceleration and rapid braking maneuvers. In acceleration maneuvers, the driver had to smoothly reach a speed of about 50 km/h from the starting position as quickly as possible. In braking maneuvers, the driver had to stop the vehicle as quickly as possible from an initial speed of 50 km/h. Measurements were carried out on test tracks with various surfaces: dry asphalt, wet asphalt, dry concrete surface, and wet concrete surface.

**Figure 1:**
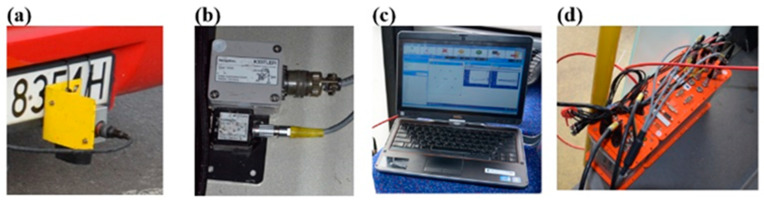
Measurement system during acceleration and braking maneuvers: (**a**) optoelectronic sensor, (**b**) acceleration sensors, (**c**) control tablet with software, (**d**) data acquisition station.

In the first stage of testing, the vehicles were equipped with a measurement system that consisted of the following:

- An optoelectronic sensor (Corrsys Datron S-350^®^ Aqua) that recorded the vehicle’s motion components, including the instantaneous speed (Figure 1a);
- A multi-functional acceleration sensor (TAA^®^) that recorded the linear acceleration, a sensor that measured the linear and angular acceleration (TANS^®^) relative to the X, Y, and Z axes (Figure 1b);
- A data acquisition station (Datron uEEP12^®^) connected with a tablet and the software (ARMS^®^) (Figure 1c,d).

The measurement system enabled data recording with a frequency of 100 Hz, thereby allowing for detailed determination of the waveforms of the maneuvers’ selected dynamic parameters.

**Figure 2:**
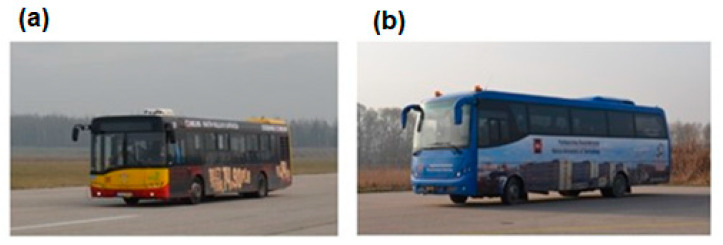
Test vehicles during acceleration and braking maneuvers: (**a**) urban bus, (**b**) coach.

The test vehicles underwent appropriate metering before the testing (Figure 2) to ensure adequate repeatability of the results. The parameters of the test vehicles used in the first stage of testing are shown in Table 1.

The aim of the first part of the study was to determine the range of limit values of longitudinal acceleration for the analyzed vehicles on various types of road surfaces. At this stage, the minimum values of the longitudinal acceleration during the rapid braking tests and the maximum acceleration values during the rapid acceleration tests were determined. The specified acceleration range can provide a reference for assessing driving style and detecting unsafe driver behavior.

The second stage of testing included a long-term recording data system installed in urban buses and coaches. The measurement system recorded vehicle velocity as a function of time and acceleration longitudinal related to time. In addition, a sensor with a GPS module was used to estimate the instantaneous position of the test vehicle. These vehicles were driven at different times of day and in various atmospheric conditions during traditional (planned) transport tasks.

Time profiles of longitudinal acceleration were recorded at this stage of testing. The measurement system used featured a sensor with a GPS module that recorded the vehicle’s instantaneous position, velocity as a function of time, and acceleration related to time. The sensor’s computer system saved the data on an SD card with a frequency of 25 Hz. It periodically archived the measurement data at a fixed time interval and designated maximum acceleration values in this interval. The data covering maximum (acceleration) and minimum (deceleration) values were used in further statistical analyses. The tests were conducted on 25 urban buses performing transport tasks for 7 consecutive days and on 8 coaches for 16 consecutive days.

## 3. Results

### 3.1. Longitudinal Acceleration Recorded during Sudden Braking

**Figure 3:**
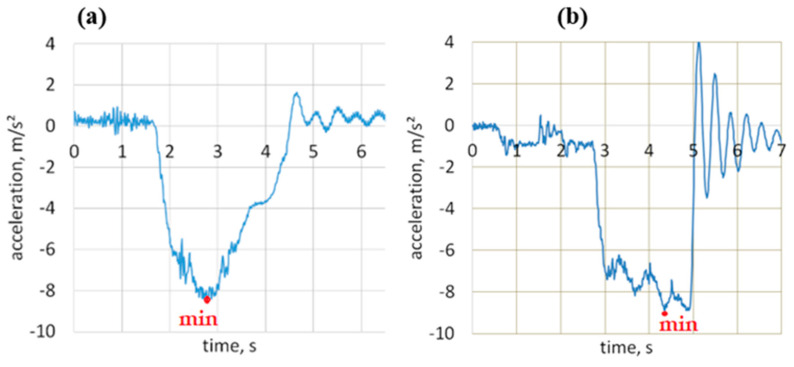
Acceleration profiles during braking for an urban bus (**a**) and a coach (**b**).

Figure 3 presents examples of deceleration profiles during braking from the initial speed of 50 km/h recorded on the track for an urban bus and a coach.

**Figure 4:**
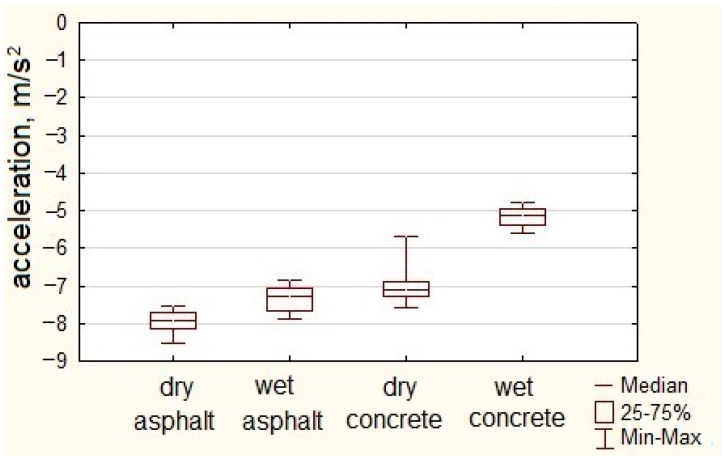
Longitudinal acceleration of urban bus during braking.

Multiple measurement cycles involved the recording of the vehicle’s motion parameters, including longitudinal acceleration profiles as a function of time, which were then used to read the minimum values. The data obtained in this way were subjected to statistical analysis. Results are shown in Figure 4.

Figure 4 shows box plots of the minimum values of longitudinal accelerations noted for an urban bus on the sudden braking maneuvers from the initial speed of 50 km/h. These were designed based on 20 series measurements.

The average of minimal negative acceleration (calculated from the minimum values) of an urban bus recorded during extreme braking maneuvers on a dry asphalt surface was −7.96 m/s^2^. The minimum value of acceleration noted during the sudden braking maneuvers was −8.54 m/s^2^. The acceleration of the urban bus noted on a wet asphalt surface was −7.35 m/s^2^. The lowest acceleration value noted during maneuvers on a wet asphalt surface was −7.88 m/s^2^.

A concrete surface is characterized by a lower grip index than an asphalt surface. The average acceleration determined based on the maneuvers performed was −7.01 m/s^2^, while the standard deviation was 0.49 m/s^2^. The lowest acceleration noted during braking maneuvers of an urban bus on a dry concrete surface was −7.58 m/s^2^. The average acceleration of an urban bus on a wet concrete surface was −5.15 m/s^2^. The acceleration lower value noted in braking on a wet concrete surface was −5.58 m/s^2^. As can be seen, the value of the standard deviation was small, which means that the obtained acceleration results were clustered around the average.

Table 2 presents selected statistical parameters of the urban bus deceleration values collected during braking maneuvers on different surfaces.

When measuring the acceleration of an urban bus in sudden braking maneuvers, a higher value was recorded on a dry asphalt surface (−8.54 m/s^2^) and the lowest on a wet concrete surface (−4.76 m/s^2^). The average value of the minimum accelerations recorded during the tests on the dry asphalt surface and wet asphalt surface differed by 8%. The average of the minimum accelerations obtained from braking tests on dry concrete surfaces was 27% greater than the average acceleration obtained from braking maneuvers on wet concrete surfaces.

**Figure 5:**
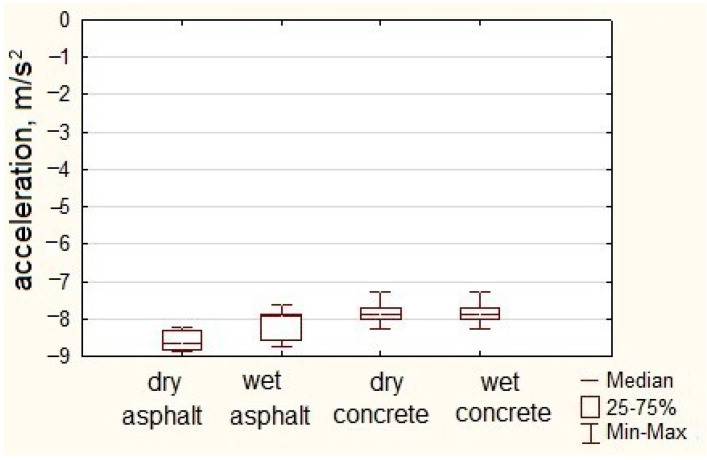
Longitudinal acceleration of coach during braking.

Figure 5 shows box plots of coach longitudinal acceleration values noted for a braking maneuver from the initial speed of 50 km/h.

The average value of acceleration determined for the series of measurements performed by the vehicle was −8.56 m/s^2^. The minimum value of acceleration noted during braking maneuvers was −8.86 m/s^2^. The average minimum acceleration value reached on the wet asphalt surface was −8.12 m/s^2^. The lowest value of acceleration achieved during braking on the wet asphalt surface was −8.74 m/s^2^.

When analyzing the braking tests of the coach on a dry concrete surface, the average minimum acceleration value was −7.84 m/s^2^ with a standard deviation of 0.29 m/s^2^. The lowest acceleration value noted in sudden braking tests of the coach on the concrete dry surface was −8.25 m/s^2^. On the concrete wet surface, the average value of minimum acceleration was −7.84 m/s^2^, and the standard deviation was 0.30 m/s^2^. The highest braking intensity on the concrete wet surface was −8.24 m/s^2^. Table 3 presents selected statistical parameters of the coach acceleration value from braking maneuvers carried out on various surfaces.

The highest value of acceleration was recorded during sudden braking maneuvers on a dry asphalt surface, which was −8.86 m/s^2^. The average of the maximum deceleration values from all the rapid braking tests on the dry asphalt surface was 5% higher than the average of the minimum acceleration values from the wet asphalt surface tests. The lowest value of deceleration was recorded during rapid braking maneuvers on a concrete surface (−7.28 m/s^2^).

### 3.2. Longitudinal Acceleration Recorded during Intense Acceleration

**Figure 6:**
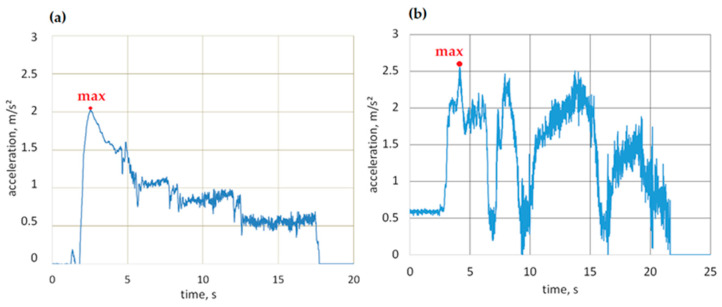
Profiles of acceleration during acceleration for an urban bus (**a**) and a coach (**b**).

Figure 6 presents examples of acceleration profiles recorded during sudden acceleration from a standing start to 50 km/h. In this case, the recorded value was the acceleration over time, which was used to read the maximum acceleration.

**Figure 7:**
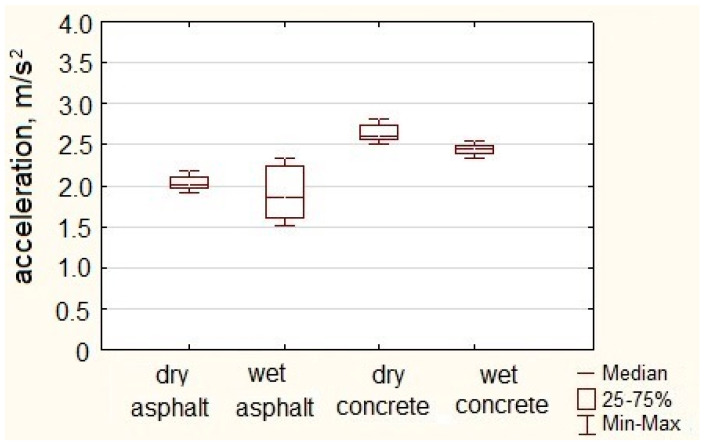
Longitudinal acceleration of urban buses during acceleration.

A statistical analysis of the obtained maximum values is presented in Figure 7, which features box plots of urban bus acceleration recorded during sudden acceleration maneuvers.

During sudden acceleration maneuvers of an urban bus on an asphalt dry surface, the average maximum acceleration value was 2.03 m/s^2^. The maximum acceleration value achieved in maneuvers on dry asphalt pavement was 2.18 m/s^2^.

During sudden acceleration maneuvers of the urban bus on a concrete dry surface, the average acceleration of the urban bus in these maneuvers was 2.64 m/s^2^. The highest acceleration value noted in rapid acceleration maneuvers of an urban bus on a concrete dry surface was 2.81 m/s^2^. On a concrete wet surface, the average value of urban bus acceleration was 2.43 m/s^2^. The highest acceleration noted in rapid acceleration maneuvers on a concrete wet surface was 2.55 m/s^2^.

Table 4 presents selected statistical parameters of the acceleration values of the urban bus obtained in the acceleration maneuvers carried out on various road surfaces.

Analyzing the maximum acceleration values achieved during the acceleration maneuver, it was noticed that the range of results for the urban bus on different surfaces was rather wide and ranged from 2.18 to 2.81 m/s^2^. The average of acceleration maximum values recorded during acceleration maneuvers on asphalt dry surfaces and asphalt wet surfaces differed by 6%.

**Figure 8:**
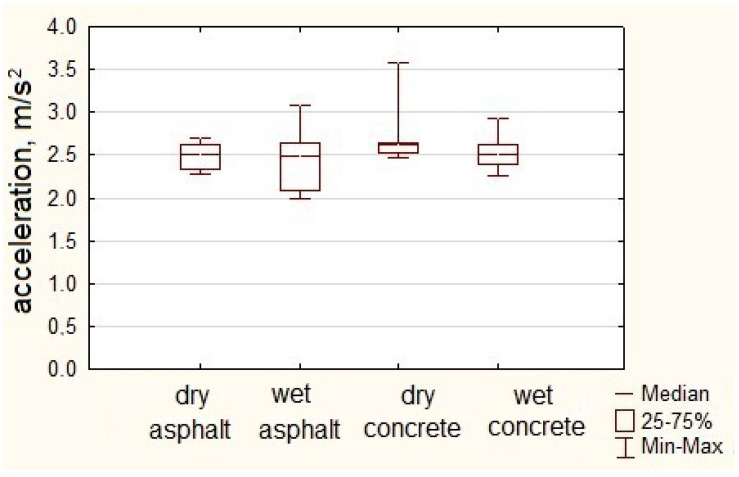
Longitudinal acceleration of coach during acceleration.

Figure 8 shows box plots of coach acceleration in sudden acceleration maneuvers up to 50 km/h.

The coach’s average maximum acceleration value during extreme acceleration maneuvers performed on an asphalt dry surface was 2.49 m/s^2^. The maximum acceleration value noted in the maneuvers was 2.70 m/s^2^. On an asphalt wet surface, the acceleration average value of the coach was 2.43 m/s^2^. The highest acceleration value in maneuvers on an asphalt wet surface was 3.08 m/s^2^.

During sudden acceleration maneuvers on asphalt dry pavement, the spread of acceleration values was 1.04 m/s^2^, and the average acceleration was 2.69 m/s^2^. The highest value of acceleration noted in sudden acceleration maneuvers on concrete dry pavement was 3.51 m/s^2^. On concrete wet pavement, the average acceleration of the coach was 2.55 m/s^2^, and the standard deviation was 0.21 m/s^2^. The highest value of acceleration in the acceleration maneuvers of the coach on the concrete wet surface was 2.93 m/s^2^.

Table 5 presents selected statistical parameters of the acceleration values obtained by the coach in the acceleration maneuvers carried out on different surfaces.

The maximum value of acceleration achieved by the coach during the acceleration maneuver on the analyzed surfaces was within the range of 2.70 to 3.51 m/s^2^. Low values of the standard deviation confirmed the repeatability of the measurements. However, the average maximum acceleration value was within the range of 2.43 to 2.69 m/s^2^. The highest on a dry concrete surface was 3.51 m/s^2^.

### 3.3. Longitudinal Acceleration of Urban Bus and Coach in Real Road Traffic Conditions

The second stage of testing involved measurements in real traffic conditions specific to both vehicle types. For urban buses, these conditions involved regular travels on various routes performed during different days of the week and at different times. Transport by coaches was performed on short and longer sections.

**Figure 9:**
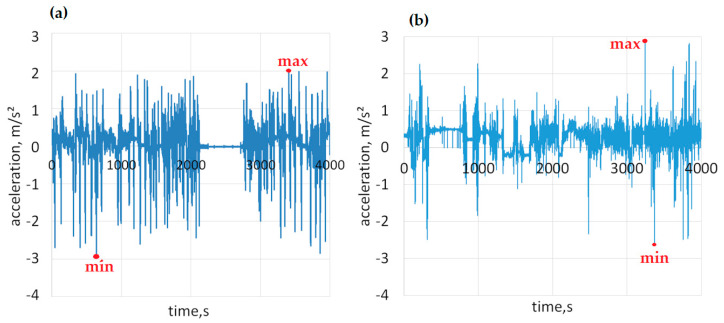
Longitudinal acceleration profiles during regular travels (**a**) for an urban bus and (**b**) for a coach.

The tests involved the recording of longitudinal acceleration in real road traffic conditions. Figure 9 presents examples of longitudinal acceleration profiles recorded for an urban bus and a coach.

With the use of the measuring equipment installed in the vehicles, the acceleration longitudinal values were continuously measured in real conditions during the regular operation of the vehicle. An analysis of the accelerations obtained provided insight into drivers’ behaviors under different road conditions. In this study, only the values of longitudinal accelerations—maximum (during acceleration) and minimum (during braking)—were presented. Statistical analysis was performed on these parameters.

**Figure 10:**
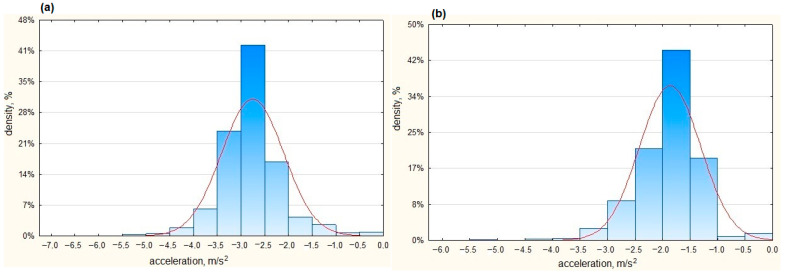
Acceleration recorded during braking in real traffic conditions (**a**) for an urban bus and (**b**) for a coach.

Positive values of the recorded acceleration corresponded to the acceleration maneuver, while negative values correspond to the braking maneuver and are identical to the deceleration presented in many publications. Figure 10 presents the ranges of negative acceleration (deceleration) recorded in real traffic conditions during braking for an urban bus and a coach.

As can be seen in real road conditions, the dominant share of maximum accelerations for the urban bus ranged from −3.0 to −2.5 m/s^2^ (42%), and for the coach from −2.0 to −1.5 m/s^2^ (44%).

Table 6 presents selected statistical parameters of the values of acceleration accumulated in real conditions during the regular operation of the coach and urban bus.

The minimum acceleration values recorded in real road traffic conditions differed. The average acceleration of urban buses during braking in real traffic conditions amounted to about −2.8 m/s^2^, while the minimum value recorded during the travel reached −6 m/s^2^. This shows that drivers relatively rarely achieve high deceleration values when braking, which may be related to the care for passenger safety. The few higher deceleration values are usually caused by traffic situations. Additionally, when we analyzed the acceleration values obtained under real conditions, it can be seen that the standard deviation reached much higher values than in the track tests. This indicates a large dispersion of values around the average values. This is due to the peculiarities of urban traffic, where the driver is forced to perform many braking and acceleration maneuvers.

For coaches, the average acceleration during braking was slightly lower and amounted to approx. −2 m/s^2^, while the standard deviation was only 0.50 m/s^2^. The minimum value of acceleration noted in braking in real traffic conditions was −5.31 m/s^2^.

**Figure 11:**
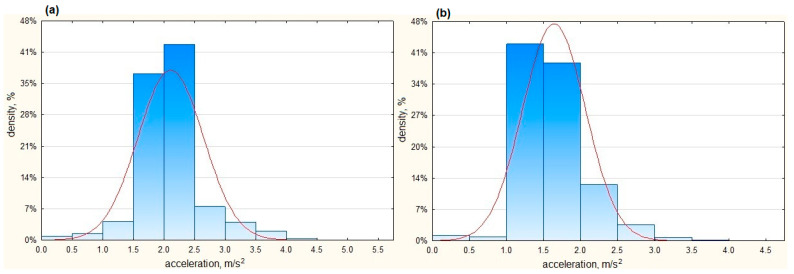
Acceleration recorded in real traffic conditions (**a**) for an urban bus and (**b**) for a coach.

Figure 11 shows the acceleration ranges of an urban bus and a coach during acceleration maneuvers.

As can be seen in real road conditions, the dominant shares of maximum accelerations for the urban bus ranged from 1.5 to 2.5 m/s^2^ (82%) and for coach from 1.0 to 2.0 m/s^2^ (82%).

Table 7 presents selected statistical parameters of acceleration values accumulated in real conditions during the regular operation of a coach and a city bus.

The average maximum acceleration values achieved by the urban buses in real traffic conditions amounted to 2.1 m/s^2^. The maximum of the acceleration values noted for an urban bus in real traffic conditions amounted to approximately 4.9 m/s^2^. The temporary high values of accelerations in the urban bus indicated dynamic phenomena during normal operations related to frequent starting and braking, e.g., when moving in a traffic jam. The average acceleration of coaches in real traffic conditions was slightly lower and amounted to 1.66 m/s^2^, and the standard deviation was 0.39 m/s^2^. The maximum acceleration noted in real traffic conditions amounted to approximately 4 m/s^2^.

## 4. Discussion

The presented study of longitudinal acceleration was separated into several parts. The first part featured sudden braking and acceleration maneuvers, and it can be observed that a change in the road surface condition and type affected the obtained acceleration values. The acceleration values obtained during braking for an urban bus demonstrate that the highest deceleration (negative value of longitudinal acceleration) was achieved on an asphalt dry surface. The maximum deceleration value recorded during the braking tests of the urban bus was 8.54 m/s^2^. The lowest deceleration value was recorded during sudden braking maneuvers on wet concrete surfaces (5.58 m/s^2^).

The deceleration achieved by a coach was substantially higher than the deceleration achieved by an urban bus during braking maneuvers on each of the analyzed surfaces. The differences are especially evident in deceleration values achieved on concrete surfaces. The maximum deceleration achieved by an urban bus on a concrete wet surface was 32% higher than the maximum deceleration achieved by a coach.

It is necessary to consider many factors in the acceleration maneuver analysis and the acceleration values achieved during this maneuver. Hydro-mechanical transmissions are usually used in urban buses, while traditional drive units are still often used in coaches. During rapid acceleration maneuvers, the differences in maximum acceleration values analyzed for each type of vehicle are no more than 0.8 m/s^2^. The highest acceleration values of the urban bus and coach were observed on the concrete surface.

It should be noted that the obtained acceleration values reflect the dynamic properties of a specific type of bus, type of road surface, and environmental conditions. However, by determining the values of extreme longitudinal accelerations in experimental conditions on the track, it can be assumed that they constitute a specific framework for the acceleration of city buses and coaches. Therefore, taking into account the aim of the project, measurements carried out in real traffic conditions are of primary importance. Obtaining by vehicles of this type of deceleration values close to the extreme values determined in this study may be a signal of a dangerous situation on the road. Similar research was presented in [40]. The results of acceleration maneuvers of rapid braking of coaches and urban buses were analyzed. During the tests of rapid acceleration to a speed of 42 km/h, the highest acceleration value was 0.37 g (3.63 m/s^2^). During braking from initial speeds of 30 km/h and 50 km/h, the highest recorded deceleration value was 0.85 g (8.34 m/s^2^).

An analysis of the longitudinal acceleration obtained during measurements in real traffic conditions demonstrated that the minimum negative acceleration values recorded for urban buses were 12% higher than the values recorded for coaches. The average value of negative acceleration noted by urban buses was 30% higher than the average value of the negative acceleration of coaches. The minimum negative acceleration recorded for urban buses during the regular course in real traffic conditions amounted to −6.01 m/s^2^, while for coaches, it amounted to −5.31 m/s^2^. The testing conducted on urban buses and coaches in real traffic conditions featured no values similar to the maximum negative acceleration values recorded during sudden braking maneuvers. This proves that during the tests there was no dangerous emergency requiring a violent reaction from the driver.

When analyzing the acceleration values obtained during tests in real traffic conditions, maximum acceleration values obtained for both types of vehicles differed by approximately 19%. The maximum acceleration recorded for urban buses amounted to 4.91 m/s^2^, while for coaches it was 3.99 m/s^2^.

The range of maximum values obtained during an urban bus’s acceleration maneuvers on tested road surfaces amounted to 2.81 m/s^2^, while the maximum acceleration value recorded in real traffic conditions amounted to 4.91 m/s^2^. As can be seen in Figure 11, high acceleration values were obtained very rarely. For a coach, the maximum acceleration value recorded in real traffic conditions amounted to 3.99 m/s^2^.

The driving style and behavior of a driver, especially a professional driver, affect the safety of other road users [41,42,43]. Depending on the driver’s driving style, traffic conditions, and other road conditions, drivers may experience different values of longitudinal acceleration. When driving in real traffic conditions, the average acceleration and deceleration values determined in the tests were 2.10 m/s^2^ and 2.78 m/s^2^, respectively. The values of longitudinal accelerations obtained for city buses presented in the paper are comparable to the results of similar tests described in the literature. The results of the study of the urban bus dynamic parameters during normal driving are presented in [44]. It was noticed that during heavy braking before traffic lights, the value of longitudinal acceleration was −0.32 g (3.14 m/s^2^). When accelerating from a traffic light, the acceleration value was 0.16 g (1.57 m/s^2^). In [45], the values of longitudinal accelerations of various vehicles during acceleration and braking at intersections with traffic lights were analyzed. The average value of acceleration of a city bus when starting from a stop before traffic lights was 0.62 m/s^2^, and the maximum value was 1.57 m/s^2^. The average deceleration when braking before traffic lights was 0.58 m/s^2^, and the maximum was 1.28 m/s^2^.

The acceleration of city buses during regular driving on the streets of Amsterdam was presented in [46]. The most common acceleration values were in the range of 1 m/s^2^ do 2 m/s^2^. The results presented in [40] show that the accelerations recorded during normal bus driving were usually less than 2 m/s^2^. During the tests, emergency situations were also recorded, and then during acceleration, acceleration values above 4 m/s^2^ were logged, and during braking the deceleration was over 8 m/s^2^.

## 5. Conclusions

The aim of the article was to determine the maximum (extreme) values of acceleration and deceleration of urban buses and coaches in rapid acceleration and braking maneuvers and the values of longitudinal acceleration during their regular driving. The presented research is part of a project aimed at creating an application for assessing the driving style of drivers and quantifying drivers using many parameters, including longitudinal accelerations. The study did not conduct comparisons of vehicles in terms of dynamics, but to learn about and present the acceleration values of these vehicles in selected maneuvers on the track and in ordinary road conditions.

Based on the results of experimental tests of vehicles on various types of road surfaces, the values show that the average maximum and minimum longitudinal accelerations obtained for the city bus are higher than for the coach. Without taking into account the type of surface, the minimum acceleration values were −7.4 m/s^2^ and −8.5 m/s^2^, respectively. The determined limit values of longitudinal acceleration during sudden acceleration and braking maneuvers carried out on the track indicate that the measurements for the analyzed vehicles were carried out correctly. This can be verified by analyzing statistical parameters, e.g., standard deviation. They reached small values not exceeding 0.5 m/s^2^ for the braking maneuver and about 0.35 m/s^2^ for acceleration. In turn, the average values determined for all types of surfaces indicated that the average maximum acceleration values during acceleration were lower for the city bus than for the coach. Without taking into account the type of road surface, the average values were 2.5 m/s^2^ and 3 m/s^2^, respectively.

This work determined the longitudinal acceleration during sudden acceleration and braking maneuvers during regular driving during normal operation of these vehicles. The values obtained in these conditions, due to the impossibility of separating the performed defensive maneuvers from normal driving, resulted in highly variable acceleration values. Taking into account the acceleration distributions obtained, one can indicate the values commonly used in road traffic and those approaching the limit values—characterizing dangerous maneuvers. One should be aware that especially in real traffic conditions, the obtained acceleration values may depend not only on the type of vehicle but also on the specificity of a given city, e.g., road infrastructure system, or road layout. Additionally, traffic regulations and the size of fines for offenses in different countries can significantly determine the tendency of drivers to drive dangerously.

The presented methodology was used in a research project, where having a sufficiently large, representative measurement base could be used to determine the driving style of drivers. In this way, it was possible to evaluate drivers on the basis of simple measurements of longitudinal acceleration values. The values obtained in measurements in real road conditions significantly exceeded the limit acceleration values set in the track tests for the braking maneuver, which may indicate the occurrence of a dangerous road event.

Drivers have their own driving styles. Additionally, the types of roads may determine the possibility of obtaining different acceleration values in various situations. The type of road surface can change quite often, so it was omitted during real traffic analyses. The recorded values of acceleration and deceleration during experimental tests on the track are treated as extreme values. If during the regular driving of the vehicle the recorded acceleration values are close to the extreme values, then it can be expected that an emergency situation may have occurred. The presented method, which used continuous measurements of accelerations, allowed the drivers of the minimum determination to achieve high accelerations. Thanks to this, it was possible to analyze the driving style of professional drivers. Appropriate actions eliminating such drivers from work or forcing them to drive safely will help to reduce dangerous situations to which passengers of these vehicles may be exposed in the future.

The presented results are only part of a project devoted to the assessment of drivers’ driving styles. Depending on the specificity of traffic, layout, and type of roads, these values may vary. Therefore, in the assumptions of the project, the fleet owner, based on the given dynamic parameters of the vehicles, can set a reference base and then, considering a specific parameter, compare the driving style of the drivers. In this way, it is possible to select drivers whose values of a given parameter differ significantly. In this paper, longitudinal acceleration was analyzed. In further research, analyses of other quantitative and qualitative parameters are planned, e.g., longitudinal acceleration, speed, overspeeding. The combination of various parameters will allow for a deeper analysis of the driver’s driving style.

## Figures and Tables

**Table 1:** Test vehicle technical parameters.

Parameter	Urban bus (Figure 2a) Solaris Urbino 12	Coach (Figure 2b) Automex Apollo
Maximum output power (kW)	224	160
Maximum torque (Nm)	1200	810
Length (m)	12.00	8.70
Mass (m)	2.55	2.42
Height (m)	3.04	3.27
Wheelbase (m)	5.90	4.22
Curb mass (kg)	10,900	7645
Total mass (kg)	18,000	10,500
Tire	Continental 275/70 R22.5	Fulda RegioForce 245/70R17.5
Number of passengers		
standing	43	30
sitting	61	-

**Table 2:** Statistical characteristics of the acceleration value in the braking maneuvers of the urban bus.

Surface/Parameter	Max m/s^2^	Min m/s^2^	Average m/s^2^	Standard Deviation m/s^2^
Dry asphalt	−7.54	−8.54	−7.96	0.27
Wet asphalt	−6.85	−7.88	−7.35	0.32
Dry concrete	−5.68	−7.58	−7.01	0.49
Wet concrete	−4.76	−5.58	−5.15	0.26

**Table 3:** Statistical characteristics of the acceleration value in the braking maneuvers of the coach.

Surface/Parameter	Max m/s^2^	Min m/s^2^	Average m/s^2^	Standard Deviation m/s^2^
Dry asphalt	−8.22	−8.86	−8.56	0.26
Wet asphalt	−7.63	−8.74	−8.12	0.38
Dry concrete	−7.28	−8.25	−7.84	0.29
Wet concrete	−7.28	−8.24	−7.84	0.30

**Table 4:** Statistical characteristics of the acceleration values in the acceleration maneuvers of the urban bus.

Parameter Surface	Max m/s^2^	Min m/s^2^	Average m/s^2^	Standard Deviation m/s^2^
Dry asphalt	2.18	1.92	2.03	0.08
Wet asphalt	2.33	1.52	1.91	0.31
Dry concrete	2.81	2.51	2.64	0.10
Wet concrete	2.55	2.21	2.43	0.09

**Table 5:** Statistical characteristics of the acceleration value in the coach acceleration maneuvers.

Parameter Surface	Max m/s^2^	Min m/s^2^	Average m/s^2^	Standard Deviation m/s^2^
Dry asphalt	2.70	2.27	2.49	0.16
Wet asphalt	3.08	2.00	2.43	0.34
Dry concrete	3.51	2.47	2.69	0.29
Wet concrete	2.93	2.26	2.55	0.21

**Table 6:** Statistical characteristics of the acceleration values accumulated under the regular driving conditions of the analyzed vehicles during braking.

Vehicle/Parameter	Min m/s^2^	Average m/s^2^	Standard Deviation m/s^2^
Urban bus	−6.01	−2.78	2.78
Coach	−5.31	−1.92	0.50

**Table 7:** Statistical characteristics of acceleration values collected in regular driving conditions of the analyzed vehicles during acceleration maneuvers.

Vehicle/Parameter	Max m/s^2^	Average m/s^2^	Standard Deviation m/s^2^
Urban bus	4.91	2.10	2.78
Coach	3.99	1.66	0.50

## Data Availability

Data are contained within the article.
